# Omega-3-Based Nutraceuticals Suppress LPS-Induced Inflammatory Responses in Primary Human Monocytes

**DOI:** 10.3390/ph19071031

**Published:** 2026-07-01

**Authors:** Thorsten Rose, Peter Schnierle, Lüder Prinzen, Bernd L. Fiebich

**Affiliations:** 1 VivaCell Biotechnology GmbH, Ferdinand-Porsche-Str. 5, D-79211 Denzlingen, Germany; thorsten.rose@vivacell.de (T.R.); peter.schnierle@vivacell.de (P.S.); 2 Ethno-Health Group B.V., Heierkerkweg 1, NL-5928 RM Venlo, The Netherlands; lueder.prinzen@ethno-health.com

**Keywords:** omega-3 fatty acids, inflammation, monocytes, cytokines, prostaglandin E2, nutraceuticals, LPS, NF-κB, immunomodulation

## Abstract

Chronic inflammation is a key contributor to the pathogenesis of numerous diseases, including cardiovascular, metabolic, and neurodegenerative disorders. Nutraceutical strategies targeting inflammatory pathways are of increasing interest, particularly those based on omega-3 fatty acids. The objective of this study was to evaluate the anti-inflammatory effects of two omega-3-based nutraceutical formulations, Omega 3 Plus and Omega 3 Orange, in primary human monocytes. Primary human monocytes were isolated from peripheral blood of a healthy donor and cultured under standardized conditions. Cells were pre-treated with different concentrations of the test formulations and subsequently stimulated with lipopolysaccharide (LPS, 10 ng/mL) for 24 h. Cell viability was assessed using the AlamarBlue assay. The release of pro-inflammatory mediators, including TNF-α, IL-1β, IL-6, MCP-1, IL-8, and prostaglandin E2 (PGE2), as well as the anti-inflammatory cytokine IL-10, was quantified using ELISA. Both formulations were well tolerated at concentrations up to 2.5%, with no significant cytotoxic effects. LPS stimulation induced a robust increase in inflammatory mediator release. Pre-treatment with Omega 3 Plus and Omega 3 Orange resulted in a significant, dose-dependent inhibition of pro-inflammatory cytokines, including TNF-α, IL-1β, and IL-6 (up to ~70% reduction). MCP-1 was moderately reduced, whereas IL-8 was only minimally affected. Notably, Omega 3 Orange exhibited a pronounced inhibition of PGE2 production (up to ~95%), while Omega 3 Plus reduced PGE2 levels by approximately 80%. Neither formulation induced IL-10 production in unstimulated cells. These findings demonstrate that both omega-3-based nutraceutical formulations exert potent anti-inflammatory effects in primary human monocytes, primarily through the inhibition of pro-inflammatory cytokines and PGE2. The strong suppression of PGE2 is consistent with a possible modulation of pathways involved in prostaglandin synthesis. These results support the potential application of such formulations in inflammation-associated conditions and warrant further mechanistic and clinical investigation.

## 1. Introduction

Inflammation is a fundamental biological response to infection and tissue injury; however, chronic or dysregulated inflammation is a key driver of numerous pathological conditions, including cardiovascular diseases, metabolic disorders, neurodegenerative diseases, and musculoskeletal degeneration [1,2,3]. Monocytes and macrophages play a central role in orchestrating inflammatory responses by producing a wide range of pro-inflammatory mediators, including cytokines such as tumor necrosis factor alpha (TNF-α), interleukin-1 beta (IL-1β), and interleukin-6 (IL-6), as well as lipid mediators such as prostaglandin E2 (PGE2) [4,5].

Lipopolysaccharide (LPS), a component of the outer membrane of Gram-negative bacteria, is widely used to model innate immune activation in vitro. LPS stimulation activates Toll-like receptor 4 (TLR4), leading to downstream activation of key inflammatory signaling pathways, including nuclear factor kappa B (NF-κB) and mitogen-activated protein kinases (MAPKs), ultimately resulting in the transcription and release of pro-inflammatory mediators [6,7].

Among these mediators, PGE2 plays a particularly important role, as it is synthesized via cyclooxygenase-2 (COX-2) from arachidonic acid and contributes to both inflammation and pain sensitization [8]. Pharmacological inhibition of COX enzymes is a major therapeutic strategy; however, non-steroidal anti-inflammatory drugs (NSAIDs) are often associated with gastrointestinal and cardiovascular side effects due to non-selective inhibition of COX-1 and COX-2 [9]. This has led to increasing interest in alternative strategies that modulate inflammatory pathways in a more balanced manner.

Omega-3 polyunsaturated fatty acids (PUFAs), particularly eicosapentaenoic acid (EPA) and docosahexaenoic acid (DHA), have been extensively studied for their anti-inflammatory properties. These fatty acids can modulate inflammatory responses through multiple mechanisms, including incorporation into cell membranes, competition with arachidonic acid for enzymatic conversion, and the generation of specialized pro-resolving mediators (SPMs) such as resolvins and protectins [10,11,12]. In addition, omega-3 fatty acids have been shown to inhibit NF-κB activation and reduce the production of pro-inflammatory cytokines in various immune cell models, including monocytes and macrophages [13,14].

While the anti-inflammatory properties of EPA and DHA have been extensively investigated, comparatively little information is available regarding the biological activity of commercially available multi-component omega-3 nutraceutical formulations. In particular, the contribution of additional bioactive ingredients and their potential synergistic effects on inflammatory mediator production remain insufficiently characterized.

In the present study, we investigated the anti-inflammatory effects of two omega-3-based nutraceutical formulations, Omega 3 Plus and Omega 3 Orange, in primary human monocytes stimulated with LPS. We specifically assessed their impact on key inflammatory mediators, including cytokines, chemokines, and lipid-derived factors, to better understand their immunomodulatory potential.

## 2. Results

### 2.1. Effects of Omega 3 Plus and Omega 3 Orange on Cell Viability

**Figure 1:**
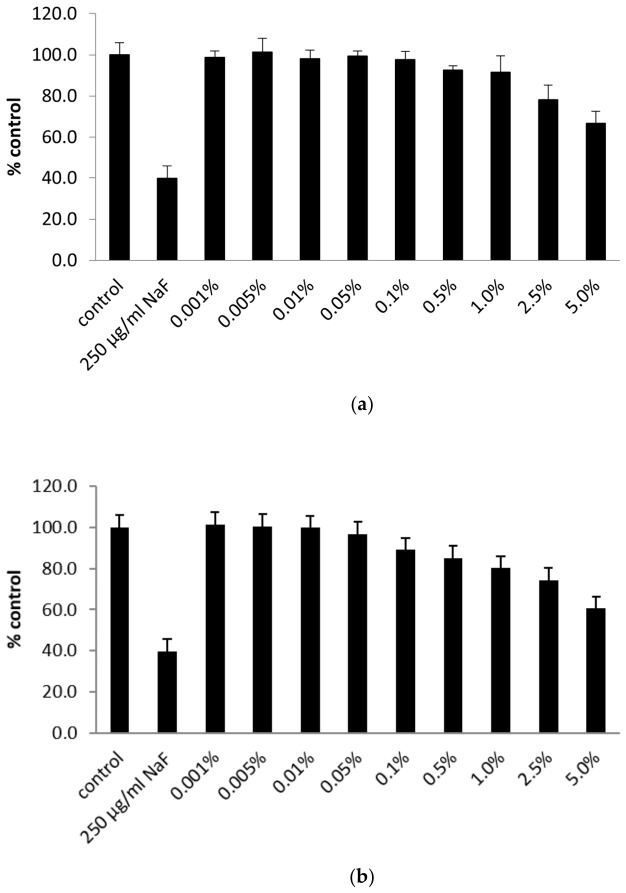
(**a**) Effects of Omega 3 Orange on cell viability of LPS-stimulated human monocytes. Cell viability was measured by AlamarBlue assay after 24 h of treatment. Values are presented as the mean ± SD of three independent experiments. (**b**) Effects of Omega 3 Plus on cell viability of LPS-stimulated human monocytes. Cell viability was measured by AlamarBlue assay after 24 h of treatment. Values are presented as the mean ± SD of three independent experiments.

The possible impact of Omega 3 Plus and Omega 3 Orange (0.001–5%) on cell viability of human monocytes was ruled out using an AlamarBlue cell viability assay. As shown in Figure 1a,b, both products did not exert cytotoxic effects, defined as 70% living cells in LPS-stimulated monocytes up to a dose of 2.5%. NaF (250 µg/mL), as a positive control, significantly induced cell death. Both products were used for further experiments in concentrations up to 2.5%.

### 2.2. Effects of Omega 3 Plus and Omega 3 Orange on Synthesis of Inflammatory Markers in Human Monocytes

**Figure 2:**
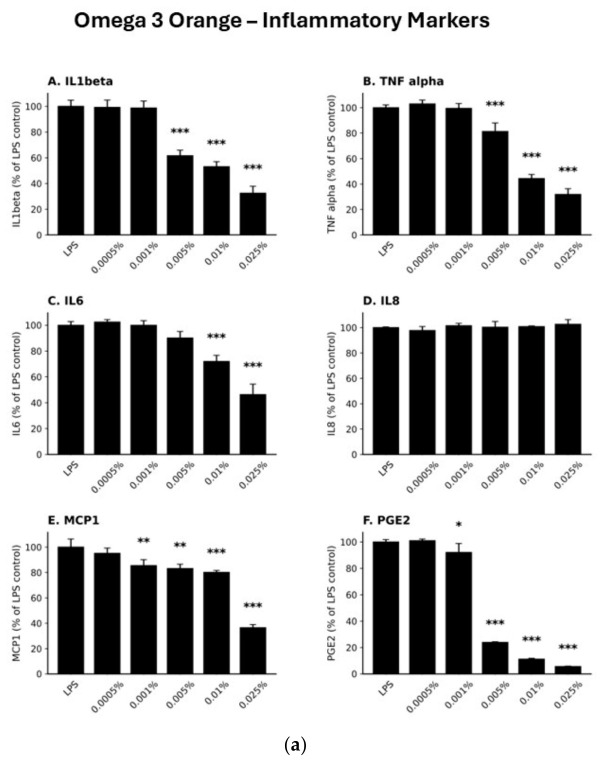

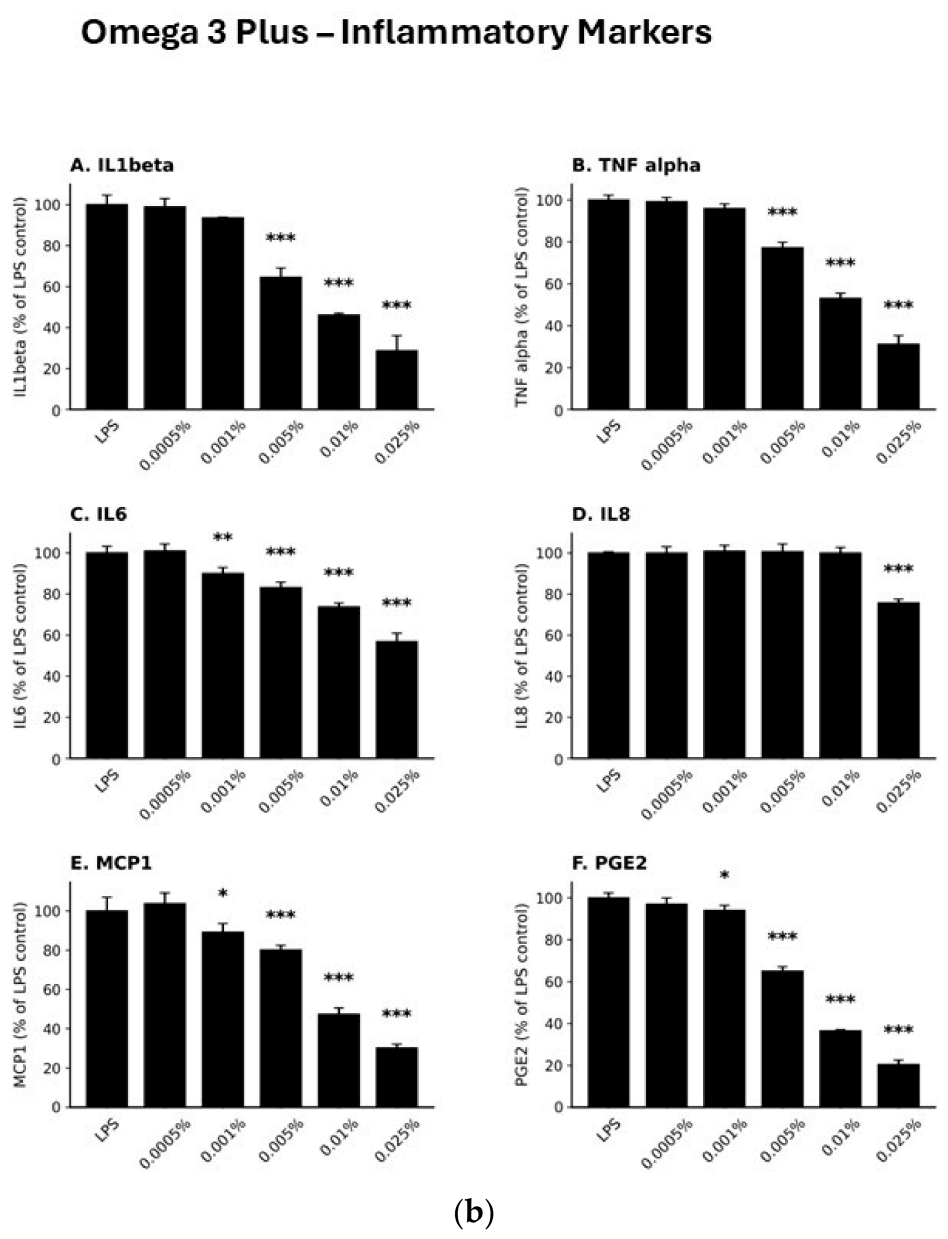
(**a**) Effects of Omega 3 Orange on the synthesis of inflammatory parameters in LPS-stimulated human monocytes. Cells were stimulated as described in Section 4. After 24 h of stimulation, supernatants were collected and the release of the various inflammatory markers was measured with ELISA. Values are presented as the mean ± SD of three independent experiments using monocytes from one donor. Statistical analysis was performed using one-way ANOVA with Dunnett’s post hoc test with * *p* < 0.05, ** *p* < 0.01, and *** *p* < 0.001 compared to LPS. (**b**) Effects of Omega 3 Plus on the synthesis of inflammatory parameters in LPS-stimulated human monocytes. Cells were stimulated as described in Section 4. After 24 h of stimulation, supernatants were collected and the release of the various inflammatory markers was measured with ELISA. Values are presented as the mean ± SD of three independent experiments using monocytes from one donor. Statistical analysis was performed using one-way ANOVA with Dunnett’s post hoc test with * *p* < 0.05, ** *p* < 0.01, and *** *p* < 0.001 compared to LPS.

Next, we analyzed the effects of Omega 3 Plus and Omega 3 Orange on protein synthesis of the cytokines IL-6, IL-1beta and TNFalpha, the chemokines IL-8 and MCP-1, and the prostanoid PGE2 in LPS-stimulated human monocytes. Briefly, monocytes were pre-treated with Omega 3 Plus and Omega 3 Orange (0.001–2.5%) for 30 min, followed by stimulation with LPS (10 ng/mL) for 24 h. The synthesis of all parameters is potently induced by LPS (Figure 2a,b).

Omega 3 Orange exhibited a highly potent inhibition of LPS-induced PGE2 release, starting at a concentration of 0.5% (Figure 2a). Maximum effects were observed at 2.5%, resulting in a highly significant inhibition of approximately 95% (Figure 2a). LPS-induced release of IL-1β and TNF-α was likewise inhibited starting at 0.5%, though less pronounced than that of PGE2 (Figure 2a). Similarly, LPS-mediated synthesis of IL-6 and MCP-1 was reduced starting at 0.5%, albeit with minor efficacy at lower concentrations (Figure 2a). For all parameters, maximum inhibition was achieved at 2.5%, reaching nearly 70% for IL-1β and TNF-α, 65% for IL-6, and approximately 50% for MCP-1. LPS-induced IL-8 was not significantly affected by Omega 3 Orange (Figure 2a).

Omega 3 Plus demonstrated a dose-dependent inhibition of LPS-induced release of IL-6, PGE2, MCP-1, IL-1β, and TNF-α starting at 0.5% (Figure 2b). Maximum effects for all parameters were reached at 2.5%, showing an inhibition of nearly 80% for PGE2, approximately 70% for IL-1β, MCP-1, and TNF-α, and 44% for IL-6. LPS-induced IL-8 was only significantly altered by the highest concentration of Omega 3 Plus (2.5%), resulting in an inhibition of 25% (Figure 2b). This effect may partially reflect the slight reduction in cell viability observed at the highest concentration tested.

## 3. Discussion

The present study demonstrates that both Omega 3 Plus and Omega 3 Orange exert pronounced anti-inflammatory effects in primary human monocytes, as evidenced by the significant inhibition of LPS-induced cytokine and mediator release. These findings are consistent with the well-established anti-inflammatory properties of omega-3 fatty acids and provide additional insight into their activity in complex nutraceutical formulations.

One of the most striking observations was the strong inhibition of PGE2 production, particularly by Omega 3 Orange, which reached up to ~95% suppression. PGE2 is a key lipid mediator generated via COX-2 from arachidonic acid and plays a central role in inflammation and pain signaling [8]. Omega-3 fatty acids such as EPA are known to compete with arachidonic acid as substrates for cyclooxygenases, leading to the production of less pro-inflammatory eicosanoids [10,15]. In addition, omega-3 fatty acids have been reported to downregulate COX-2 expression, thereby further reducing PGE2 synthesis [16]. The pronounced inhibition observed in this study suggests that the tested formulations may modulate both substrate availability and enzymatic activity within the eicosanoid pathway.

The stronger inhibition of PGE_2_ compared with cytokine production may indicate that the formulations affect lipid mediator pathways particularly efficiently. Besides competition of EPA and DHA with arachidonic acid, additional ingredients such as curcuma-derived compounds may contribute to the observed effect through modulation of inflammatory enzyme systems.

In addition to effects on lipid mediators, both formulations significantly reduced the release of key pro-inflammatory cytokines, including TNF-α, IL-1β, and IL-6. These cytokines are central regulators of inflammatory cascades and are primarily controlled by transcription factors such as NF-κB [6].

Previous studies have demonstrated that omega-3 fatty acids can interfere with NF-κB signaling [13,17]. Whether this mechanism contributes to the effects observed in the present study remains to be determined experimentally.

The moderate inhibition of MCP-1 (CCL2), a key chemokine involved in monocyte recruitment, further supports the anti-inflammatory profile of the formulations. Reduced MCP-1 levels may contribute to decreased immune cell infiltration in inflamed tissues, which is relevant for chronic inflammatory conditions such as atherosclerosis and neuroinflammation [18].

Interestingly, neither formulation significantly affected IL-8 production, except for a modest effect at higher concentrations of Omega 3 Plus. IL-8 is primarily involved in neutrophil recruitment and may be regulated differently from other cytokines, suggesting that the formulations selectively modulate specific inflammatory pathways rather than inducing broad immunosuppression.

Another notable finding is the lack of induction of IL-10, a key anti-inflammatory cytokine. This suggests that the observed effects are primarily driven by suppression of pro-inflammatory signaling rather than activation of anti-inflammatory feedback mechanisms. Such a profile may be advantageous, as it indicates targeted modulation of inflammation without overstimulation of immune-regulatory pathways.

The overall anti-inflammatory effects observed in this study are consistent with previous reports demonstrating that omega-3 fatty acids reduce inflammatory responses in human immune cells [13,14]. However, the magnitude of inhibition, particularly for PGE2, suggests that additional components within the formulations may contribute synergistically. These could include antioxidants, vitamins, or plant-derived compounds, which are known to modulate oxidative stress and inflammatory signaling pathways [19,20]. Recent evidence indicates that nutraceutical compounds may modulate inflammatory and oxidative stress pathways through complementary mechanisms involving cellular redox regulation and immune signaling [20].

From a translational perspective, the ability of these formulations to suppress key inflammatory mediators in primary human monocytes highlights their potential relevance for a wide range of inflammation-associated conditions. Chronic low-grade inflammation is implicated in cardiovascular diseases, neurodegenerative disorders, and musculoskeletal degeneration, all of which involve monocyte/macrophage activation [1,2,3]. Therefore, nutraceutical interventions targeting these pathways may represent a complementary strategy to conventional pharmacological approaches.

Future studies should aim to further elucidate the underlying molecular mechanisms, including the involvement of NF-κB, MAPK pathways, and COX-2 expression. In addition, investigation of oxidative stress-related pathways, such as Nrf2 signaling, may provide further insight into the broader biological activity of these formulations. Curcumin-containing nutraceuticals have been reported to modulate Nrf2-dependent cellular defense pathways and inflammatory signaling, providing a plausible avenue for future mechanistic investigation [21].

## 4. Materials and Methods

### 4.1. Materials

Omega 3 orange (150 mL) (Product No. EH0495 (with vitamin D; EAN 4262454610495) and Omega 3 plus (150 mL) (Product No. EH7446 (EAN 4260271527446) (Ethno Health Group BV, Venlo, The Netherlands)) are vegan omega-3 formulations based on *Schizochytrium* algae oil rich in DHA and EPA. Omega 3 orange contains Sacha-Inchi oil and rapeseed oil, whereas Omega 3 plus contains hemp seed oil and rapeseed oil. Both products additionally contain curcuma, vitamins D/E, and a proprietary herbal extract complex.

### 4.2. Primary Monocytes

Monocytes were extracted from the whole blood obtained from one healthy volunteer after written informed consent in accordance with institutional guidelines and the Declaration of Helsinki. According to local regulations, no additional ethical approval was required for the use of anonymized buffy coats from healthy donors. Monocytic cells (University Hospital of Freiburg, Germany) were enriched following a standardized protocol (gradient preparation, Lymphocytes separation medium, PAN Biotech, P04-60125, Aidenbach, Germany) using completely endotoxin-free cultivation. Using 50 mL tubes, 25 mL of the separation medium was loaded with 25 mL of blood (buffy coats). The gradient was established by centrifugation at 1800 rpm, 20 °C for 40 min with slow acceleration and deceleration. Peripheral blood mononuclear cells in the interphase were carefully removed and re-suspended in 50 mL pre-warmed phosphate-buffered saline (PBS) (Pan Biotech, P04-36500, Aidenbach, Germany), followed by centrifugation for 10 min at 1600 rpm and 20 °C. The supernatant was discarded and the pellet washed in 50 mL PBS and centrifuged as described above. The pellet was then re-suspended in 50 mL RPMI-1640 low-endotoxin medium supplemented with 10% human serum (Hexcell, Berlin, Germany, SP2080). After counting the number of cells in a particle counter (Euro Diagnostics, Krefeld, Germany), cells were seeded in 24-well plates for enzyme-linked immunosorbent assay (ELISA) (2.2 × 10^6^ cells/well) or 96-well plates for cell viability testing and incubated at 37 °C with 5% CO_2_. The medium and the non-adherent cells (lymphocytes) were removed and fresh RPMI-1640 medium containing 1% human serum was added. Enriched monocytes were thus ready to be used for the experiments.

### 4.3. Cell Viability Assay

An AlamarBlue assay (Thermo Fisher Scientific, Bonn, Germany) was performed to identify the possible cytotoxic effects of both products. Cytotoxicity was analyzed by AlamarBlue staining. Cells were seeded on 96-well cell culture plates and incubated for 24 h in 5% CO_2_ at 37 °C. On the following day, the medium was changed and after at least 1 h, cells were treated without or with different concentrations of both products for 24 h (0.001–5%). NaF (250 µg/mL, Sigma-Aldrich, Taufkirchen, Germany) was used as a positive control to induce cell death. Then, cells were washed once with 100 μL PBS and 100 μL of medium-AlamarBlue-Mix (90% medium, 10% AlamarBlue, DAL1025, Thermo Fisher Scientific, Bonn, Germany) was added to each well. The plate was incubated at 37 °C for 2 h in a humidified 5% CO_2_ atmosphere, and the color reaction was determined using a 96-well plate reader (excitation 544 nm, emission 590 nm).

### 4.4. Determination of Cytokine and Chemokine Release

ELISA was used for quantitative protein analysis directly in the adherent cell’s supernatant. Human monocytes were left untreated or pre-treated with different concentrations of Omega 3 Plus or Omega 3 Orange for 30 min. All formulations were tested simultaneously using monocytes isolated from the same healthy donor to minimize inter-individual biological variability and to allow direct comparison between treatments. Concentrations were selected based on preliminary range-finding and cytotoxicity experiments and were chosen to cover a broad concentration range while maintaining acceptable cell viability. After that, LPS was added for 24 h except for the negative control. Afterwards, the supernatants were collected and centrifuged at 1000× *g* for 2 min at 4 °C. Commercially available ELISA kits for chemokines and cytokines (Bio-Techne/R&D Systems Europe, Ltd., Abingdon, UK) and PGE2 (Cayman, distributed by BioMol, Hamburg, Germany) were used to investigate the effects of Omega 3 Plus and Omega 3 Orange on the protein synthesis of TNF-α, IL-6, IL-8, IL-1beta, MCP-1, and PGE2 as instructed by the manufacturer. Briefly, 96-well plates (Thermo Fisher Scientific, Bonn, Germany) were coated with the respective capture antibodies. On the following day, the plates were blocked and washed, and standards and supernatants were prepared based on the pre-test-determined dilutions and added into the respective wells. Detection antibody, streptavidin-HRP, and stop solution were added step by step as advised by the manufacturer. The absorbance of the wells was read at 450 nm using the MRXe Microplate reader (BMG Labtech, Offenburg, Germany). Data were normalized to LPS control and presented as a percentage of change in cytokine and chemokine levels.

### 4.5. Statistical Analysis

All statistical analyses were carried out using GraphPad Prism 8.0 (Prism 8 software, GraphPad software Inc., San Diego, CA, USA). Raw values were converted to the percentage of LPS (10 ng/mL) considered as 100%. The data are represented as the mean ± SD of at least three independent experiments. Multiple comparisons data were analyzed using one-way ANOVA with Dunnett’s post hoc test. The level of significance was set at * *p* < 0.05, ** *p* < 0.01, *** *p* < 0.001, and is indicated in the figures.

## 5. Conclusions

The marked suppression of prostaglandin E2 together with a consistent reduction in pro-inflammatory cytokines highlights the potential of omega-3-based nutraceutical formulations to modulate key inflammatory pathways in human immune cells. The pronounced effect on PGE2 is particularly noteworthy given its central role in inflammation and pain, consistent with a possible interaction with pathways involved in prostaglandin synthesis.

These findings support the concept that complex omega-3 formulations may represent a targeted and physiologically balanced approach to inflammatory regulation, distinct from classical pharmacological inhibitors. The observed activity in primary human monocytes underscores their translational relevance and potential applicability in inflammation-driven conditions.

Future studies should further delineate the underlying molecular mechanisms and evaluate the clinical relevance of these effects, particularly in the context of chronic low-grade inflammation and related pathologies.

## 6. Limitations

Despite the robust anti-inflammatory effects observed in this study, several limitations should be acknowledged. First, a pharmacological anti-inflammatory reference compound was not included, which limits direct comparison with established anti-inflammatory agents. Second, a further limitation is that all experiments were performed using monocytes obtained from a single healthy donor. Nevertheless, this established ex vivo human monocyte assay has previously been evaluated using monocytes isolated from several independent buffy coats, demonstrating comparable inflammatory response patterns and supporting the robustness and reproducibility of the experimental model [22]. Consequently, donor-to-donor variability could not be assessed and the findings should therefore be interpreted as preliminary observations requiring confirmation in additional donors. Third, the experiments were conducted exclusively in vitro using primary human monocytes, which, although highly relevant, do not fully reflect the complexity of in vivo inflammatory responses. Fourth, the precise molecular mechanisms underlying the observed effects were not directly investigated, and future studies should address signaling pathways such as NF-κB, MAPKs, and COX-2 expression. Fifth, the exact contribution of individual components within the tested formulations remains unclear, as the study focused on the overall activity of the products rather than isolated compounds. Finally, the concentrations used in vitro may not directly translate to physiological conditions, highlighting the need for further pharmacokinetic and clinical investigations.

## Data Availability

The original contributions presented in this study are included in the article. Further inquiries can be directed to the corresponding author.

## Abbreviations

The following abbreviations are used in this manuscript:

DHA	Docosahexaenoic acid
ELISA	Enzyme-linked immunosorbent assay
EPA	Eicosapentaenoic acid
IL	Interleukin
LPS	Lipopolysaccharide
MAPK	Mitogen-activated protein kinase
MCP-1	Monocyte chemoattractant protein-1
NF-κB	Nuclear factor kappa B
PGE2	Prostaglandin E2
PUFA	Polyunsaturated fatty acid
TLR4	Toll-like receptor 4
TNF	Tumor necrosis factor alpha
